# Clinical Lecture on a Case of Pott’s Disease of the Upper Cervical Vertebræ

**Published:** 1877-09

**Authors:** S. W. Gross

**Affiliations:** Lecturer on Clinical Surgery at the Jefferson Medical College


Clinical Lecture on a Case of Pott's Disease of the Upper Cervical Vertebræ.—*By S. W. Gross, M.D., Lecturer on Clinical Surgery at the Jefferson Medical College.—Gentlemen:* You will notice in this girl, who is fifteen years of age, deformity of the nape of the neck, and a peculiarity in the position of the head, which is slightly flexed, advanced forwards, and inclined somewhat to the right side. Her mother states that she detected the swelling four months ago; but I take it for granted that the disease upon which it depends had existed for some months previously. In the morning, when she rises from her bed, she is able to hold the head more nearly in an upright position and has more control over its movements, than later in the day. At this time also the muscles in front of the neck are relaxed. At about noon, she complains of pain, which radiates down the shoulders and arms, and the head is fixed in the constrained and rigid position in which you now see it. Pressure upon the top of the head, rotation, and attempts to extend the head greatly aggravated the pains; but they are much relieved when she goes to bed, although they recur two or three times during the night, awakening her from her sleep. Two months ago, she also suffered from pains which extended as high as the vertex on both sides of the occipital region.

The prominence on the posterior surface of the neck, which is the seat of an unnatural amount of heat, and is tender on pressure, extends from the occiput to the spinous process of the fifth vertebra. There is very slight projection of the separate spines, so that the deformity presents less of an angular appearance than is met with in tubercular disease of the column lower down.

Two weeks ago, this girl's father died of pulmonary phthisis: so that her trouble is a legacy transmitted by her parent. It is a beautiful illustration of Pott's disease, or tubercular disease of the spine, occurring, however, higher in the column than is generally the case, the dorsal region being the one most frequently affected. It would be interesting to determine the existing cause of the affection; but this I have been unable to do, and I can elicit no history of injury. Suppression of the cutaneous perspiration or disorder of the digestive apparatus would be sufficient to produce it, since in this disease there is always a latent spark lodged somewhere in the system, and ready at any moment to be aroused into activity by the slightest cause. I must confess that I belong to the school of pathologists who maintain that no one can have such a disease as this, or any other of those which are termed tubercular or strumous, without there being a predisposition to it.

The first four cervical vertebræ are here involved in strumous inflammation. Such an affection may be simulated by rheumatic or common inflammation of the vertebral ligaments; but in such cases, while pain would result from pressure upon the head, reflected painful sensations would not be experienced in the occipital region, the shoulders, and the arms. The value of reflected, or so-called sympathetic pains, in the diagnosis of the disease, is not sufficiently appreciated by the mass of the profession, and to those of you who feel interested in pursuing this topic, I can earnestly recommend the highly instructive and interesting "Lectures on Rest and Pain," delivered by Mr. John Hilton, an eminent surgeon of London. In cases of tuberculosis of the spine, peripheral pain will almost invariably point out the seat of the lesion weeks before the deformity has established the diagnosis. Hence, when a child is brought to you suffering pain somewhere upon the surface, it should be stripped, and the course of the painful nerve be traced back to its point of emergence from the intervertebral foramen.

In the case before us, one of the earliest symptoms, and one which clearly indicated spinal trouble, was bilateral pain over the occiput, which reached as high as the vertex, and followed the distribution of the great occipital nerves, which are branches of the second cervicals and emerge from the spinal canal between the atlas and axis vertebræ. The bodies of these bones being invaded by inflammation, the nerves in the intervertebral foramen become irritated, and express their irritation by pain which is referred to their peripheral distribution. Subsequently, pain was experienced in the shoulders and arms; and this is readily accounted for when we consider that the lower trunks of the superficial plexus, which are distributed to the tissues over the scapula and clavicle, are involved in the morbid action. You may ask, what has the superficial plexus to do with the pain in the arms? Between the fourth cervical nerve and the fifth cervical nerve, which forms apart of the brachial plexus, there is a communicating branch; and it is along this that the sensation of pain is reflected down the arm.

The pain is relieved at night, or whenever the patient assumes the recumbent posture. During the day, while the girl is moving about and sitting up, the bodies of the carious vertebræ are pressed against one another, as is shown by the flexed and advanced position of the head. The pressure is produced by the weight of the head, and increased by the contraction of the muscles attached to it. The sterno-mastoids are only slightly contracted; but there are a number of others which flex the head and are supplied by branches of the cervical plexus. Two in particular are involved; they are the rectus capitis anticus major, its upper and middle portions, and the longus colli. They arise from the transverse processes and bodies of the cervical vertebræ, and are inserted, one in front of the foramen magnum of the occipital bone, and the other into the tubercle on the anterior arch ef the atlas. The weight of the head, pressing on the bodies of the vertebræ, irritates the nerves; the nerves of sensation react on the nerves of motion, and the muscles are stimulated to contraction, thereby causing still greater pressure, and keeping up the pains. A similar effect is produced in coxalgia, where the adductor and pectineus muscles are firmly contracted. When our patient goes to bed, the weight of the head is removed, the irritation of the nerves subsides, and the muscles relax. On rising in the morning, no muscular resistance is offered to the nodding motion of the head, and it can be brought into a nearly vertical position. After two or three hours, however, the weight again stimulates the muscles to contraction, and the flexion and pain recur.

The pains felt at night are due to reflex muscular action. During the day, while the brain is on the alert, the muscles which steady the vertebral column are under the control of the will, and are kept constantly in a contracted state to prevent motion; but during sleep the irritation of the exposed filaments of nerves in the cancelli of the bones is transmitted only to the medulla oblongata, the muscles are involuntarily thrown into contraction, and pain is produced by the inflamed surfaces of bone being brought violently together.

The prominence of the back of the neck is due not only to the absence of the bodies of the vertebræ and the flexed position of the head, but also in part to the projection of the spinous process of the axis vertebra. When the head is flexed the atlas vertebra slides forward, making the axis prominent. The greater part of the swelling is, however, due to the deposition of inflammatory new material between and around the posterior segments of the vertebræ. It is placed there for the purpose of forming a splint to keep the parts at rest. The contraction of the muscles answers the same purpose. Nature is doing her best to cure this girl, but will not entirely succeed, and in her present condition she is in danger. Owing to the softening of the atlas vertebra, the transverse ligament may become torn away by an incautious movement; the odontoid process would then be forced backward into the medulla, and produce sudden death.

There was a time when this disease could have been met so as to have prevented deformity. It should have been attened to when the reflected pains gave warning of the impending mischief, or before there was much destruction of the bones and the intervertebral disks. Then, with proper care, a large amount of the now inevitable distortion could have been avoided. All that we can do in the way of treatment is to check the progress of the disease and prevent further deformity. The patient's hands are cold and clammy, showing that the circulation in the outskirts of the system is imperfect, and her appetite is poor. Hence I shall order her two grains of quinine, fifteen drops of the tincture of the chloride of iron, and five drops of the tincture of nux vomica, to be taken every eight hours, along with a nourishing diet, of which an abundance of fresh milk and bread shall form a prominent feature. With a view to equalizing the circulation, her mother will be instructed to sponge her off with the tepid water every morning, and to follow this by friction with a salt towel, which is made by dipping a towel in a strong solution of common salt and allowing it to dry in the sun.

You need scarcely be told that the further progress of the disease can only be arrested by keeping the parts perfectly at rest and free from pressure. If you have carefully weighed the symptoms, you will have observed that you have only to follow out what nature prompts you to do. When the patient is recumbent, the vertebræ are relieved of the weight of the head, and a night's rest is sufficient to relax the contracted muscles and relieve the pain, which is a source of so much suffering. Hence it must be evident to you that rest in the recumbent posture is imperatively demanded in this case. To make it effectual, the patient should lie on a hair mattress, the head and neck being supported by a small pillow of sand or small grain, either of which will mould itself to the inequalities of the surface; while the movements of the head are still further restrained by short bags of sand placed on each side and reaching from the top of the head to the shoulders. In this position she will be bathed and fed, and pass her excretions, and not be allowed to rise for any purpose whatever. To be of service, recumbency should be strictly enforced, and be prolonged for months if it be necessary, or until such a time as the vertebræ shall have become consolidated by bony material, which will be indicated by freedom from pain on practising the manœuvres which now elicit it.

A common idea, but, according to my experience, a most erroneous one, is that children bear recumbency badly; that they waste, lose their appetites, and not infrequently die, in consequence of their close confinement. Some cases of joint-disease go on from bad to worse under any course of treatment; but I speak advisedly when I say that recumbency causes children, in the majority of instances, to grow fat and strong. It is the pain which induces emaciation and even death; and there is no apparatus with which I am acquainted that entirely relieves pain if the patient be permitted to walk about during the acute stage of the affection. Should, however, it be deemed advisable to give the patient the benefit of fresh air, it may be carried out in a cuirass, which includes the head, made of wire and well padded. So soon as pain is not elicited by pressing upon or rotating the head, the child may be allowed to get on its feet, provided the spine, weakened by disease, be relieved of the weight of the head. For the richer class of patients, the best support is that of Mr. Darrach, of Orange, New Jersey, in which the weight of the suspended head is transferred to the trunk and pelvis by means of a steel rod, attached above to a padded plate which accurately fits the occipital convexity, and below to a plate fixed on a corset made of rawhide. As the corset is moulded on a plaster cast of the body it accurately fits all the inequalities of the surface; and as it is perforated by numerous round openings it is extremely light, while freedom of the respiratory movements is provided for by fastening the corset in front with elastic bands. To the occipital plate is attached a band which runs around the sides and front of the head, and from which another band passes under the chin. In the poorer classes, the gypsum bandage of Dr. Sayre, in the folds of which a support for the head is embraced, answers a most admirable purpose.—*Medical Times.*

